# Extracorporeal Photopheresis (ECP) and the Potential of Novel Biomarkers in Optimizing Management of Acute and Chronic Graft vs. Host Disease (GvHD)

**DOI:** 10.3389/fimmu.2020.00081

**Published:** 2020-01-31

**Authors:** Matthew Mankarious, Nick C. Matthews, John A. Snowden, Arun Alfred

**Affiliations:** ^1^Department of Haematology, Sheffield Teaching Hospitals NHS Foundation Trust, Royal Hallamshire Hospital, Sheffield, United Kingdom; ^2^Department of Photopheresis, The Rotherham NHS Foundation Trust, Rotherham, United Kingdom

**Keywords:** extracorporeal photopheresis, GvHD, immunomodulation, biomarkers, apoptosis, dendritic cells

## Abstract

As the use of hematopoietic stem cell transplantation (HSCT) has become a more widespread and effective treatment for hematological malignant and non-malignant conditions, the need to minimize the harmful effects of graft- vs.-host disease (GvHD) has become more important in achieving good outcomes. With diagnosis of GvHD reliant on its clinical manifestations, research into biomarkers for the diagnosis, progression, and even for the prediction of disease, is imperative to combating the high levels of morbidity and mortality post-HSCT. Despite the development of novel treatment approaches to GvHD, corticosteroids remain the standard first-line treatment, with immunosuppressant therapies as second-line options. These strategies however have significant limitations and associated complications. Extracorporeal Photopheresis (ECP) has shown to be effective and safe in treating patients with symptomatic GvHD. ECP has been shown to have varied effects on multiple parts of the immune system and does not appear to increase the risk of relapse or infection in the post HSCT setting. Even so, ECP can be logistically more complex to organize and requires patients to be sufficiently stable. This review aims to summarize the potential role of biomarkers to help guide individualized treatment decisions in patients with acute and chronic GvHD. In relation to ECP, robust biomarkers of GvHD will be highly useful in informing patient selection, intensity and duration of the ECP schedule, monitoring of response and other treatment decisions alongside the concurrent administration of other GvHD therapies. Further research is warranted to establish how GvHD biomarkers are best incorporated into ECP treatment pathways with the goal of tailoring ECP to the needs of individual patients and maximizing benefit.

## Introduction

Hematopoietic stem cell transplantation (HSCT) has become an established routine treatment for hematological malignancies, with over a million transplants having taken place across five continents (1). However, a major limiting factor of this curative treatment is the development of graft vs. host disease (GvHD), which is a key cause of morbidity and mortality to patients following allogeneic HSCT (2), where control of GvHD is central to optimizing long-term outcomes. The therapeutic action of HSCT relies on the graft vs. leukemia (GvL) effect, which therefore makes systemic immunosuppression for GvHD prevention and treatment undesirable (3). Effective prevention and treatment of GvHD is therefore a challenging balance between targeting GvHD whilst maintaining the GvL effect.

Increased understanding of the pathophysiology of GVHD has driven strategies to enable earlier diagnosis, alter management and apply new therapeutic interventions (4).

## Graft vs. Host Disease (GvHD)

GvHD presents as two distinct clinical syndromes, acute (aGvHD) and chronic (cGvHD).

### Acute GvHD

Acute GvHD is characterized by a marked inflammatory reaction thought to be mediated by donor T lymphocytes recognizing the host tissue as non-self relatively soon after transplantation. The most commonly seen sites of involvement are the skin (maculopapular rash), liver (bile duct damage and cholestasis with hyperbilirubinemia), and the gastrointestinal tract (vomiting, anorexia, and severe diarrhea) (5, 6). aGvHD is reported to manifest in 30–50% of allogenic-HSCT recipients, of which 14% experience severe aGvHD (grades III-IV on the modified Glucksberg-Seattle criteria), associated with poor outcomes (6). In practice aGvHD is diagnosed clinically, supported by exclusion of differentials and histological confirmation, with risk of development related to donor-recipient histocompatibility (7, 8). Biopsy and histological examination is a crucial part of the work up and can be logistically challenging to obtain before starting treatment but is very useful in confirming the diagnosis and in disease staging (7, 8). Corticosteroid treatment remains standard first line therapy though there is no standard effective second line treatment for those failing steroids (9).

### Chronic GvHD

Chronic GvHD is reported to affect 30–40% of patients receiving allogeneic HSCT (10). The pathophysiology of cGvHD comprises of complex pathways involving both T and B cells, the mechanisms of which are yet to be fully understood (11). The myriad clinical manifestations of cGvHD can make diagnosis and monitoring response to treatment challenging. Following NIH 2014 working group recommendations, diagnosis is made clinically based on presence of at least one diagnostic manifestation or at least one distinctive manifestation supported by relevant tests such as histology, which should differ from the hallmark signs of aGvHD (dermatitis, enteritis and cholestasis) with recommendations made also to standardize monitoring and response assessment (12). Treatment of cGvHD comprises first line of corticosteroids, usually prednisolone, often in combination with a calcineurin inhibitor (13). Around 50% of patients with established cGvHD respond to steroids, but only 20% are living without disability after 4 years (14). Steroids with adjuvant therapies have also shown to have no overall benefit when compared to steroids alone (14). Further therapies of cGvHD include inhibition of B cell signaling (Ibrutininb), Inhibition of T cell signaling (Ruxolitininb), Depletion of B cells (Rituximab), T reg sparing therapy (Sirolimus), and T reg expansion (ECP, IL2) (4).

### Overview of Biomarkers in GvHD

Significant progress has been made in identifying and validating biomarkers for GvHD (15). Based on the 2014 NIH consensus (16), these biomarkers have been investigated for diagnostic, prognostic, and predictive use, and additionally to assess treatment response. Given the heterogeneity of the condition and differing clinical practice the consensus statement also highlighted the need for any potential biomarkers to be validated by at least two independent cohorts prior to investigation in clinical setting including trials and patient management. Such prediction of risk and prognostic information would allow the stratification of patients according to their individual risk and a tailoring of treatment regimens and prophylaxis to reduce the severity of tissue damage. Diagnostic biomarkers could allow pre-emptive treatment to be started before clinical manifestation of GvHD and further monitoring with biomarker-mediated assessment of treatment response.

#### Biomarkers in aGvHD

The most validated serum biomarker for aGvHD (Table 1) is ST2 (suppression of tumorigenicity 2). A serum level of ST2 measured on Day 14 post transplantation has been shown to be associated with significantly increased risk of aGvHD, including treatment-resistant aGvHD with increased non-relapse mortality (NRM) and predictive of transplant related mortality (TRM) (17, 18). An additional study described levels of ST2 to be predictive of NRM within 1 year (19). Due to its functional relationship to tissue damage and immune function, ST2 has been considered the best candidate biomarker to indicate severity and prognosis of aGvHD (20). The role of ST2 in GvHD pathogenesis has been explored further with monoclonal antibody blocking of soluble ST2 in the peri-transplant period showing protection against GvHD whilst preserving GvL activity (21). Regenerating Islet-derived 3-alpha (Reg3α) has been validated as a prognostic and diagnostic biomarker specific to gastrointestinal aGvHD. Increased serum Reg3α levels post-transplantation have shown to indicate an increased incidence of severe aGvHD, thought to be caused by the destruction of GI paneth cells and impaired epithelial function (22), which was also indicative of poor prognosis following treatment (23). A 2 biomarker panel based algorithm combining ST2 and Reg3α levels measured 7 days post-transplant has been shown to stratify patients on the basis of NRM into two distinct high risk and low risk groups (24). It has to be borne in mind that such biomarkers can also be elevated in other pathologies associated with an inflammatory milieu in this period such as thrombotic microangiopathy, cytokine release syndrome, mucosal inflammation and idiopathic pneumonia. These confounding variables may have an impact on the rate of false positive results (19, 25, 26). Similarly, T-cell immunoglobulin mucin-3 (TIM3), thought to exacerbate aGvHD severity, has been shown to be useful identifying patients with higher risk of severe GvHD and mortality, and additionally shows potential in predicting failure of corticosteroid treatment (28, 29). sTNFR1 (soluble tumor necrosis factor receptor-1) and IL-6 have been found to be valuable in predicting incidence of severe aGvHD and NRM (19), with sTNFR1 additionally predicting treatment failure (29). Biomarkers have also been studied with respect to the affected organ with specific targets including skin (Elafin) (30).

**Table 1:** Summary of biomarkers in acute GvHD.

**Biomarker**	**Study**	**Use**	**Cohort**	**Findings**
ST2	Vander et al. (17)	Predictive	First 3m post-transplantation in 673 patients, at start of GvHD treatment in 381	ST2 levels measured at the initiation of therapy for GVHD and during the first month after transplantation improved risk stratification for treatment-resistant GVHD and death without relapse after transplantation
	Ponce et al. (18)	Predictive	Day 28 samples from 113 cord blood transplant patients	ST2 was the only biomarker associated with grades II-IV and III-IV aGVHD and transplant related mortality
	McDonald et al. (19)	Predictive	149 GvHD patients across 2 cohorts, 167 GvHD-free patients	ST2 was found to be useful in predicting more severe GvHD and non-relapse mortality.
Reg3α	Zhao et al. (22)	Diagnostic	28 allogeneic transplant patients who developed GI GvHD symptoms	Reg3α serum levels rose in systematic circulation as GVHD progressively destroyed Paneth cells and reduced GI epithelial barrier function
	Cai et al. (23)	Diagnostic/ Prognostic of GI aGvHD	103 allo-HSCT patients, serum collected before and after transplantation and following GvHD treatment	Increased plasma Reg3α level after transplantation suggests the incidence of grades III-IV GI-aGVHD. The high level of plasma Reg3α in patients with grades III-IV GI-aGVHD after the immunosuppressive treatment for 4 weeks indicates a poor prognosis.
	Shin et al. (27)	Predictive	Discovery set of 5 aGVHD patients and 5 controls, compared to an independent validation set of 89 patients	Plasma-derived protein biomarkers including Reg3α can be used to predict aGVHD and NRM before the onset of clinical manifestations.
TIM3	Abu Zaid et al. (28)	Predictive	Multicenter study with uniform GVHD prophylaxis, conditioning regimen, and donor source, explored correlation biomarkers with outcomes in 211 patients	High plasma TIM3 at day 28 correlated with 2-year non-relapse mortality in multivariate analysis and overall survival
	McDonald et al. (29)	Predictive	165 patients after 14 days of glucocorticoid therapy to evaluate associations with treatment failure and non-relapse mortality	Clinical findings (serum bilirubin, skin GVHD) and plasma biomarkers (TIM3, ST2, sTNFR1) can predict failure of GVHD treatment and NRM. However, inadequate positive predictive values for identifying high-risk GVHD cohorts
sTNFR1IL-6	McDonald et al. (19)	Predictive	149 GvHD patients across 2 cohorts, 167 GvHD-free patients	Levels of IL6 and sTNFR1 had utility in predicting development of grade 3–4 GVHD. sTNFR1 predicted non-relapse mortality within 1 year after transplantation

Lower GI(Reg3α,TIM3) (22) and liver (HGF, KRT18) (31) IL-6 has been implicated as a pro inflammatory agent in the context of GvHD and blocking this appears to have a dampening effect on GvHD (32). C-reactive protein (CRP) as a surrogate marker for IL6 is a routinely available inflammatory marker, has been shown to be a good indicator of aGvHD risk in a PRISM compliant meta-analysis (33), and a 2012 study has shown potential of fecal calprotectin and α-1-antitrypsin as biomarkers (34), which again are markers readily available in routine practice. An alternative approach to soluble biomarkers has been to look at changes in patterns or counts of cellular mediators as predictive biomarkers of aGvHD. One of the earliest targets of aGvHD is the vascular endothelium resulting in endothelial GvHD (35). In a prospective sequential analysis of 90 allo-HSCT patients circulating endothelial cells (CEC) counts increased 1–2 weeks before and peaked at onset of aGvHD (36). Conversely, CEC counts returned to pre-transplant baseline after treatment response. Another method is detailed monitoring and statistical analyses of multiple subsets of lymphocytes by flow cytometry; in a study of 50 HSCT patients aGvHD development was significantly associated with increased frequencies of central memory CD4 T cells (Tcm) and memory B-cells pre-HSCT and by increased frequencies of memory, naïve, T-reg and recent thymic emigrant (RTE) T-cell subsets at aGVHD onset (37).

Although aGVHD is primarily mediated by alloantigen-specific donor lymphocytes, the initial trigger for disease development is thought to be the activation of antigen-presenting cells (APCs) by danger signals from damaged tissues and pathogen-associated molecular patterns (PAMPs) through a class of highly evolutionarily-conserved pattern recognition receptors called Toll-like receptors (TLRs) (38). In a prospective study of the expression of all 9 human TLRs in a cohort of 34 allo-HSCT patients, development of aGVHD correlated with high monocyte and T-cell expression of TLR5 and low expression of TLR1 and TLR9 (39). TLR5 recognizes flagellin, a component of the flagella of motile bacteria, including intestinal bacteria (40), which translocate to the blood following damage to the intestinal mucosa (41). High expression of TLR5 might lead to increased responses to TLR5 agonists leading to enhanced stimulatory capacity and pro-inflammatory cytokine production by APCs and increased activation and proliferation of effector T-cells (49). Similarly, ligation of TLR1 (which recognizes bacterial lipopeptides) and TLR9 (which recognizes viral and bacterial DNA) both stimulate pro-inflammatory cytokine production (50). How low levels of expression of these TLRs is associated with aGvHD is unclear, but may be linked to cross-regulation of TLRs (51).

#### Biomarkers in cGvHD (Table 2)

Compared to aGvHD, less has been accomplished in the validation of biomarkers for cGvHD; however several candidates of note have substantial evidence for their potential use. B-cell activating factor (BAFF) is one such candidate, and one of the first biomarkers associated with cGvHD. Increased BAFF has been linked with the pathogenesis of cGvHD, through increased abnormal B-cell survival and BAFF levels were shown increased in chronic GVHD patient sera (42, 43). A recent study confirmed the correlation between onset of cGvHD and increased soluble serum BAFF (44), and a further study found patients without cGvHD showed gradually decreasing BAFF levels as B cell numbers increased after myeloablative conditioning and significantly different BAFF/B cell ratios at 3 months post-HSCT in patients who subsequently developed cGVHD (45). A 2016 study across two cohorts aimed at identifying diagnostic and prognostic biomarkers for cGvHD resulted in a panel of 4 proteins (ST2, CXCL9, matrix metalloproteinase 3, and osteopontin) shown to indicate prediction of cGvHD diagnosis, and additionally prognostic risk stratification post-HSCT (46). This study showed strength in the reproducibility of its results across a second cohort, with samples from eight different sites used (46). With ST2 shown to be a valid biomarker for aGVHD and a target for monoclonal antibody blocking (21), additional therapeutic benefit might be derived in cGvHD. The CXCR3 chemokine receptor has interferon-inducible ligands CXCL9 and CXCL10, which have previously been shown to have a role in trafficking CXCR3^+^ T cells toward the peripheral tissues (59). These ligands have been shown to be useful as potential cGvHD biomarkers. CXCL9 levels were shown in one prospective, multicentre study to be associated with cGvHD (28), and similarly found to be elevated in cGvHD plasma when compared to healthy or non-cGvHD controls (47). CXCL10 was also shown to be elevated in cGvHD plasma (47), and was the only biomarker investigated to meet the criteria of Kariminia et al. for replication as a clinical biomarker for the diagnosis of cGVHD (48).

**Table 2:** Summary of biomarkers for chronic GvHD.

**Biomarker**	**Study**	**Use**	**Cohort**	**Findings**
BAFF	Allen et al. (42)		*Ex vivo* analyses of peripheral B cells from 51 patients with and without cGVHD 1-year post HSCT	Exogenous BAFF treatment amplified cell size and survival in B cells from patients
	Ahmed et al. (43)	Diagnostic	Two center study, biomarkers evaluated pre-HSCT and serially post-transplant, with time-matched control samples from patients without GVHD	BAFF levels were increased in chronic GVHD patient sera
	Rozmus et al. (44)		Cohort of 44 post-HCT patients with cGVHD and 63 time-matched recipients without cGVHD	Onset of cGVHD was associated with higher soluble BAFF levels
	Jacobson et al. (45)	Prognostic	Prospectively monitored 412 patients in the first year after allogeneic transplantation	Patients without cGvHD showed gradually decreasing BAFF levels as B cell numbers increased after myeloablative conditioning Significantly different BAFF/B cell ratios at 3 months post-HSCT in patients who subsequently developed cGVHD
4 protein panel (ST2, CXCL9, MMP3, Osteopontin)	Yu et al. (46)	DiagnosticPrognostic	Compared pooled plasma samples obtained at matched time points after HSCT (median, 103 days) from 35 patients with cGVHD and 18 without cGVHD. Second verification cohort of 172	Panel with an AUC of 0.89 and significant correlation with cGVHD diagnosis, severity, and non-relapse mortality. In a second verification cohort, this panel distinguished patients with cGVHD (AUC, 0.75), and measured at day +100 could predict cGVHD occurring within the next 3 months with an AUC of 0.67 and 0.79 without and with known clinical risk factors Measurements at diagnosis or day +100 may allow patient stratification according to risk
CXCL9	Abu Zaid et al. (28)		A prospective, multicenter study with uniform GVHD prophylaxis, conditioning regimen, and donor source, measured biomarkers from plasma samples collected in 211 patients	CXCL9 levels above the median were associated with chronic GVHD compared with levels below the median in a time-dependent proportional hazard analysis
	Hakim et al. (47)		Analysis of gene expression in circulating monocytes	Found elevated levels of CXCL9 in cGvHD plasma, as compared to levels in normal control or non-cGvHD plasma
CXCL10	Kariminia et al. (48)		Two independent replication cohorts (total of 134 cGVHD cases and 154 controls	CXCL10 strongly correlated in both replication sets when GVHD cases and controls were evaluated for several clinical covariates, and their impact on biomarkers was identified by univariate analysis
	Hakim et al. (47)		Analysis of gene expression in circulating monocytes	Found elevated levels of CXCL10 levels in cGvHD plasma, as compared to levels in normal control or non-cGvHD plasma

## Extracorporeal Photopheresis (ECP) as an Immunomodulatory Treatment Modality for GvHD

ECP is a cell-based immuno-modulatory treatment whereby the buffy coat of peripheral blood, containing leukocytes and platelets, is separated, treated to a photosensitizing agent (8-methoxypsoralen) and exposed to UVA light and re-infused back to the patient. This treatment was initially reported by Edelson who published on the use of ECP in the treatment of erythrodermic cutaneous T-cell lymphoma (CTCL) in context of a multicentre trial (60). It is a mature treatment modality and for over 20 years has been used for chronic and acute graft vs. host disease (cGVHD) and solid organ transplant rejection (61, 62).

### Clinical Application of ECP in Acute GvHD

There is no standard second line treatment for patients who are either refractory to first line steroids or have steroid dependent aGvHD (63). There is an unmet need for a modality of treatment which offers immunomodulation rather than immune suppression as a way of reducing the effect of GvHD. Given its potential impact on T cell mediated responses, ECP has been studied as a treatment option in aGvHD where there is an immunological donor T cell response to host alloantigens. In a pioneering phase II study, 59 patients with acute steroid-refractory GVHD grades II to IV were treated with ECP weekly and response and long-term survival were assessed (64). Eighty-two percent of patients with cutaneous involvement, 61% with liver involvement and 61% with gut involvement achieved complete response (CR). Among responders the survival probability was 59% compared to 11% in patients not responding completely. Further at 4 years the transplant related mortality was significantly lower for patients achieving a CR to ECP (14 vs. 73%) with an overall survival (OS) at 4 years 59 vs. 11% in those achieving CR. Similar responses were noted in another study of 27 patients for steroid resistant GvHD (65) with a suggestion of better response at the early initiation of ECP in steroid resistant disease. This was confirmed in another report with higher response rates when treatment was started within 35 days of onset of aGvHD (66). ECP has also been studied in relation to the use of anti-cytokine therapy for aGvHD with a multicentre comparative analysis showing significantly higher response in the ECP arm compared to etanercept or inolimumab arm with patients receiving ECP showing a survival advantage (67). Looking at the response to ECP in a systematic review, Abu-Dalle et al. (68) showed aGvHD overall response rates to ECP were 69% across 323 patients in 9 studies (95% confidence interval 0.34 to 0.95) (49). Highest response was seen in cutaneous aGvHD, followed by gastrointestinal. The American Society of Blood and Marrow Transplantation have developed recommendations for treatment of aGvHD based on evaluation of 29 studies (9). In regard to ECP there was no increase in overall rates of infection particularly viral reactivations, which can be a major concern with ongoing immunosuppressive treatment though it did not specify any single agent in the second line setting. This view is also echoed by the guidelines issued by the British Society for Blood and Bone Marrow Transplantation (BSBMT) (69). Similar recommendations have been made by the Italian scientific societies the Italian Society of Hemapheresis and Cell Manipulation (SIdEM) and the Italian Group for Bone Marrow Transplantation (GITMO) in their best practice recommendation for the use of ECP in acute and chronic GvHD in adults and children (70). Currently ECP is considered a potential treatment option for patients with aGvHD grades II-IV who are steroid refractory, steroid dependent or steroid intolerant in the HSCT setting and for solid organ transplant rejection (61, 62).

### Clinical Application of ECP in Chronic GvHD

Since the initial report of its use in 1994 to successfully treat cGvHD (71), ECP has been shown as an effective and recommended treatment for cGvHD, including steroid refractory GvHD (72, 73). In a review of both prospective and retrospective studies in the secondary treatment of cGvHD published between 1990 and 2011, ECP was the most frequently studied therapy (74). Flowers et al. reported a phase 2 randomized controlled prospective study of ECP treatment in cGvHD (72). The study compared standard treatment alone with the addition of ECP in cutaneous cGvHD. The proportion of patients who had at least a 50% reduction in steroid dose and at least a 25% decrease from baseline in TSS was 8.3% in the ECP arm at week 12 and 0% in the control arm (*P* = 0.04). The non-blinded investigator assessment of skin complete or partial responses revealed a significant improvement in favor of ECP (*P* < 0.001). A limitation of this study was that skin score was the main focus of assessment and physicians were aware of study assignment. Progressive improvement in symptoms and increased steroid sparing effect was seen in longer ECP treatment of 24 weeks, reported by Greinix et al. in a follow up study (75). A recent randomized control prospective study with 60 patients compared addition of ECP to standard of care in the first line setting using the NIH 2015 criteria for diagnosis and response assessment. ORR at week 28 was 74.1% (ECP arm) vs. 60.9% (control arm). Furthermore, patients in the ECP arm tolerated the treatment well and crucially maintained quality of life (QoL) whilst there was a decline in QoL scores in patients in the standard care arm (76). In a prospective trial evaluating the efficacy of ECP in both skin and visceral cGvHD (77), Foss et al. enrolled 25 patients with extensive, steroid-refractory cGvHD. 20 patients had improvement in cutaneous GVHD and six had healing of oral ulcerations. Steroid sparing or discontinuation of immunosuppressive medications was possible in 80% of patients with similar response rates between patients receiving treatment weekly vs. fortnightly treatments. A review of 27 studies including 725 adults treated with ECP for steroid-resistant, intolerant, or dependent cGvHD of (61). The mean response rate for cutaneous cGvHD 74% (reported in 23 studies) hepatic cGvHD was 62% (15 studies), 60% for ocular cGvHD (4 studies), and 62% for mucosal cGvHD (reported in 12 studies). Pierelli et al. reviewed 23 studies reporting on 735 patients treated with ECP for steroid-resistant, -intolerant, or -dependent cGvHD (70). As a whole, overall and complete responses were observed in 64 and 35% of cases with cutaneous involvement and in 56 and 27% with hepatic cGvHD, respectively. Overall response rate was also 47 to 57% in oral mucosa and gastrointestinal tract cGvHD. High response rates, near 50%, were also reported in children with ocular involvement. In 2012, Del Fante et al. reported on a retrospective analysis of 102 patients with cGVHD treated with ECP over a 14 year period, assessing whether the NIH consensus classification better predicted survival and response to ECP (78). The study found no correlation between response and NIH clinical subtype, number, or degree of organ involvement, and found no response in patients with lung involvement. A retrospective multicentre evaluation of ECP as second line treatment for acute and chronic GvHD reported a response in at least 80% with long term survival of at least 50% of the cases (79). Abu Dalle et al. in their systematic review evaluating the efficacy of ECP treatment in steroid refractory or steroid dependent GvHD, similarly suggest organ-specific response to be higher in cutaneous, gastrointestinal, hepatic, and oral mucosa, with very limited effect of ECP on pulmonary cGvHD (68).

An important therapeutic effect of ECP in cGvHD is steroid reduction whilst controlling GvHD thereby having an impact on the morbidity and mortality related to prolonged immunosuppression (77, 80, 81). ECP has also been shown to maintain responses to viral infection and does not increase the risk of relapse (82, 83) QoL is an important measure of outcome for patients undergoing allogeneic HSCT comparable with scores reported for systemic sclerosis, systemic lupus erythematous, and multiple sclerosis (84). In a prospective study evaluating the effect of ECP on clinical response and QoL in cGVHD using two validated questionnaires, there was significant improvement in both cGVHD symptoms scale and DLQI scores in patients who completed 6 months of ECP (85).

### Immunological Mechanisms of ECP Action

Over 30 years after ECP was invented its definitive modes of action remain elusive. While ECP may be considered one of many apoptotic cell therapies being exploited for inducing immunotolerance to auto- and alloantigen, its lack of immunosuppressive effect and proven clinical effectiveness against CTCL as well as GVHD is both confounding and intriguing. Exposure to 8-MOP/UVA induces cross-linking of DNA, triggering a series of apoptotic events including loss of mitochondrial membrane potential, caspase activation and phosphatidylserine exposure (86). The flipping of phosphatidylserine from the inner plasma membrane leaflet to the outer surface is one of an array of “eat-me” signals recognized by professional phagocytes such as macrophages and dendritic cells which facilitates the specific removal of dead, damaged, and dying cells (87). The removal of apoptotic cells by phagocytes is termed “efferocytosis” meaning “to bury” and is essential for tissue and immune system homeostasis (88, 89). ECP has direct effects on lymphocytes, NK cells, neutrophils, and monocytes with neutrophils and NK cells being most readily affected while monocytes and myeloid dendritic cells have been reported to show the greatest resistance (90, 91). The data for the effects of ECP on monocyte cell death are conflicting. While some groups report that monocytes are as susceptible to ECP-induced apoptosis as other PBMC (92–94), others show marked survival (95–97) or showed no greater levels of cell death than untreated controls (98, 99). The reported preferential survival of monocytes may be facilitated by integrin-mediated survival signals generated through interaction of monocytes with plasma proteins bound to plastic surfaces in the ECP instrument, which subsequently directed differentiation into monocyte-derived dendritic cells (100). While neutrophils constitute the largest fraction of leukocytes treated and ultimately rendered apoptotic by ECP (90), infusion of ECP-treated leukocytes has been reported to rapidly mobilize patient neutrophilic myeloid-derived suppressor cells (MDSC) into the circulation (101). Functionally, these MDSC could suppress Th1 and Th17 responses and longitudinal studies showed a relationship between therapeutic response to ECP and progressive increase in peripheral blood MDSC frequency.

**Figure 1:**
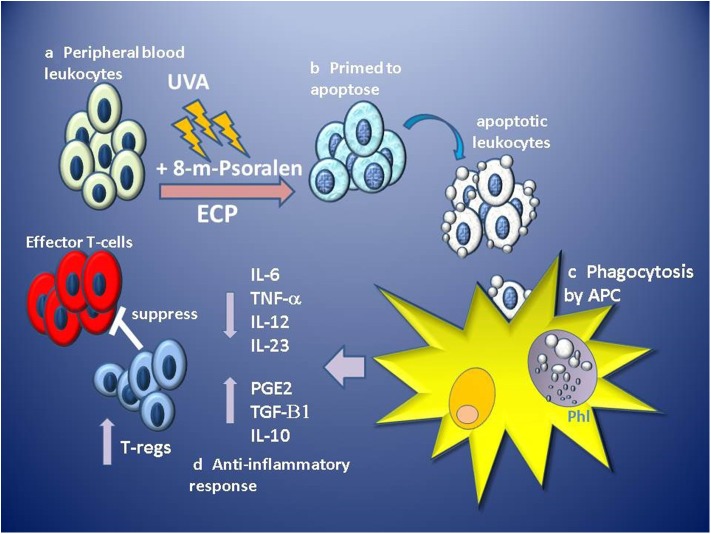
Stages of proposed primary hypothesis for mechanism of immunomodulation of GVHD by ECP. (a) Apheresed peripheral blood leukocytes are separated from red blood cells and concentrated before exposure to 8-methoxypsoralen (8-m-psoralen) and photoactivated by UV-A light (UVA). (b) ECP-treated leukocytes now primed to die by apoptosis are infused into the circulation. (c) Apoptotic leukocytes are recognized, engulfed and phagocytosed by antigen presenting cells (APC: macrophages and dendritic cells) in phagolysosomes (Phl). (d) Recognition of apoptotic cells induces an anti-inflammatory tolerogenic response by APCs resulting in lower production of pro-inflammatory cytokines IL-6, IL12, IL-23, and TNFα and induces production of anti-inflammatory IL-10, TGF-β1, and prostaglandin E2 (PGE2). Tolerogenic APCs promote the priming and expansion of regulatory T-cell (T-regs), which suppress the function of alloantigen-specific effector T-cells involved in GVHD.

ECP results in the functional suppression and subsequent deletion of large numbers of pathogenic leukocytes from the circulation, however, it is thought that since only 5–10% of circulating leukocytes are directly affected, this is unlikely to be the primary mechanism of effect (102). Instead, it is the indirect, wider and sustained immunomodulatory effect of the uptake and processing of ECP-treated cells on the effectors of disease, which confers therapeutic benefit. ECP primes massive numbers (> 2 × 10^9^–dependent on size and state of the patient) of leukocytes for cell death which are infused in high density (>20 × 10^6^ cells/ml) back into the patient through venous return within 4–6 min, but *in vitro* analysis suggests apoptotic features are not induced until at least 4 h after ECP treatment (90). Tracking of infused radiolabelled ECP-treated PBMC and neutrophils in patients revealed that both were detected in the lungs, spleen and liver within 10 min, but had different patterns of migration, with PBMC being initially retained in the lungs in greater quantity than neutrophils, but then subsequently trafficking to the liver and spleen (103), suggesting that ECP-treated leukocytes retain homing ability for at least a few hours post-infusion. These observations are consistent with *in vivo* tracking studies of apoptotic cells in murine models where intravenously infused apoptotic cells are phagocytosed by macrophages and dendritic cells located in the lung, liver and spleen (104, 105). The uptake of apoptotic cells by macrophages induces a suppression of IL1-β,IL-6, IL-12, and TNF-α proinflammatory cytokine production while inducing the secretion of TGF-β1 and PGE-2 (106). Similarly, dendritic cell uptake of apoptotic cells induces a tolerogenic phenotype characterized by low levels of expression of costimulatory molecules, suppressed production of proinflammatory cytokines and enhanced production of anti-inflammatory IL-10 producing an APC with low capacity to stimulate the generation of T-cell effectors, instead, generating the priming of TGF-beta 1-dependent FoxP3 regulatory T-cells (105) (Figure 1).

In an *in vivo* model of ECP treatment of acute GVHD, weekly infusion of splenocytes from an allogeneic donor with acute GVHD, given after HSCT, strongly enhanced survival, and reversed established GVHD symptoms (107). The mechanism of protection was dependent on donor-derived CD25hi FoxP3 T-regs found in increased numbers in the spleen and was coupled with a decrease in splenic CD8^+^ T-cell effectors. GVHD is characterized by a lack of circulating T-regs, which can potentially exert regulatory effects on T-cell effectors and DCs at all stages of GVHD as well as facilitating tissue repair through the secretion of factors such as amphiregulin (6, 108, 109). T-regs mediate immunotolerance and part of the therapeutic effect of immunosuppressive drugs such as rapamycin and glucocorticoids is mediated through the promotion of induced T-regs (110, 111). However, while ECP facilitates immunotolerance there are conflicting data regarding the role of T-regs in ECP immunodulation of GVHD. While some groups report an expansion of circulating numbers of T-regs (92, 112–114), others show expansion, but no correlation to response in terms of steroid tapering or disease score (115). In a randomized prospective trial of ECP for cGVHD there was no significant change in the frequency of circulating T-regs or skin-homing T-regs (116). Similarly, in a trial combining ECP with low-dose IL-2, which has shown promise in expanding T-regs in cGVHD patients (117), there were no differences in the absolute counts of circulating T-regs between ECP-responders and non-responders although both showed marked T-reg expansion in the first few weeks of starting IL-2 treatment (118). Such observations in patients do not readily fit a model of ECP being primarily mediated through the induction of T-regs and other experimental data challenge this paradigm in the understanding of autologous ECP- treatment of ongoing inflammatory disease. A more recent *in vivo* model has shown that infusion of ECP/PUVA-treated cells from an allogeneic healthy donor failed to provide protection or reverse acute GVHD development, whereas splenocytes from an allogeneic donor of the same genetic background with acute GVHD provided significant protection (119). Further, in an *in vivo* model of ECP-modulation of rheumatoid arthritis, only ECP-treated splenocytes from arthritogenic donors could suppress inflammation, whereas those from healthy donors had no significant effect (120). Such observations suggest that supply of apoptotic cells alone is insufficient to control ongoing severe inflammatory diseases. It is of note that most of the studies using apoptotic cell therapy to prevent allograft rejection use donor cells that are from healthy donors and are thus from an immune environment that is in the steady state and the cells are resting or non-activated. In contrast, ECP for treatment of GVHD is autologous and many PBMC are activated. While apoptotic resting cells are tolerizing, activated or damaged cells can be immunogenic (121). This is illustrated in an *in vivo* delayed type hypersensitivity model where infused apoptotic resting naive CD4 T-cells induced tolerance, but apoptotic activated CD154^+^ CD4 T-cells were immunogenic and licensed DCs to recruit and prime CD4 T-cell effectors (122). Hannani et al. have observed that ECP- treated HLA-DR^+^ activated lymphocytes from GVHD patients die quicker than their non-activated counterparts (123) and have proposed a novel model where these would be preferentially phagocytosed and their antigens processed and presented before the slower dying non-activated fraction (124). Through being activated these are potentially immunogenic and might license DCs to prime anti-clonotypic cytotoxic T-cells to target and delete the alloantigen-specific pathogenic clones mediating GVHD. This model is compatible with ECP being free of general immunosuppression and can accommodate the apparent contradiction of ECP being effective for both immunotolerizing against GVHD and immunostimulatory against CTCL (124). Indeed, recent data suggests that tolerogenic and immunogenic effects can be potentially exerted by different cell types in the same ECP-treated sample since apoptotic neutrophils down-regulated LPS-induced DC and macrophage inflammatory cytokine production and reduced overall APC activation. In contrast, co-culture with apoptotic CD3 T-cells activated both APCs and enhanced LPS-induced proinflammatory cytokine production, particularly of TNF-α, coupled with enhanced APC allostimulatory capacity (125).

### Biomarkers in Relation to ECP Treatment of GvHD (Table 3)

An early study by French et al. (52) was one of the first on biomarkers for ECP response in GvHD. The authors investigated whether circulating clonal T cells in peripheral blood and clonal T cell receptor γ (TCRγ) rearrangement, could be linked to response to ECP, as was previously demonstrated in cutaneous T cell lymphoma (CTCL) (53). Using fluorescent based PCR and capillary electrophoresis, peripheral blood samples of 27 patients post-allogenic HSCT were analyzed for TCRγ gene rearrangement. Seventeen of the patients studied had extensive cGvHD and 10 were without GvHD. TCRγ gene rearrangements and amplified clonal T cell populations were found in 60% of the patients without cGvHD and in 76.5% of patients with cGvHD, compared to 0% of the healthy controls. Twelve of the cGvHD patients received ECP treatment, 8 of which had significant response. It was found that all the patients who responded to ECP had amplified clonal T cell populations and those who did not respond to treatment did not. It was therefore concluded that expanded clonal T cell populations in the patients with cGvHD before treatment increased significantly the probability of cutaneous response to ECP. A subsequent study by Kuzmina et al. (54) investigated levels of immature B lymphocytes in 49 patients with moderate and severe cGvHD, measuring immature CD19^+^CD21^−^ B cells and memory CD19^+^CD27^+^ cells before ECP and 6, 12 and 21 months into ECP treatment. Patients who showed no response to ECP after 6 months had significantly higher proportions of immature CD19^+^CD21^−^ cells prior to ECP treatment, compared to patients with complete and partial response. The proportions of memory CD19^+^CD27^+^ cells prior to ECP were not significantly different between the groups, however there was a significantly higher ratio of CD21^−^ to CD27^+^ cells before treatment in patients showing no response. A 2010 study reported by Akhtari et al. (55) investigated correlation of response to ECP with patients' baseline circulating dendritic cells (DCs) and T lymphocytes. Twenty-five patients with cGvHD were treated with ECP, with 2 procedures on consecutive days every week for the first 2 months, then every other week for 2 months, followed by once monthly. Baseline number of myeloid and plasmacytoid DC precursors, and CD4^+^ and CD8^+^ T lymphocytes, were measured using flow cytometry. The study concluded that patients who responded to ECP had higher baseline circulating DCs and T cells, which can predict response to ECP in cGvHD patients. The study noted that apart from a decrease in CD4^+^ cells in responsive patients, there was no significant change in T cell of DC populations over the year following ECP treatment. Following the focus of Kuzmina et al. on B lymphocytes as predictive biomarkers, Whittle and Taylor (56) investigated serum BAFF measurements in 46 cGvHD patients undergoing ECP treatment and demonstrated the potential use of BAFF as a biomarker to predict treatment response in cutaneous GvHD. BAFF levels after 1 month of ECP predicted response at 3 and 6 months. Patients with BAFF concentrations of <4 ng/mL showed decreased skin GvHD and complete resolution in 11 patients and those with high BAFF concentrations showed worsened skin GvHD at 6 months and resolution in only 1 patient. Subsequent measurement of BAFF after 3 months of treatment was reported to predict probability of maintaining improvement at 6 months. The study reported BAFF concentration only to correlate to skin GvHD but full responders to ECP in skin GvHD also had more improvements in other organs than those who did not. Bertani et al. (57) focused on the T lymphocyte population including CD3+. They reported a 2015 retrospective study on the response of steroid-refractory cGvHD to ECP, linking CD3^+^ lymphocyte count in harvested peripheral blood during ECP procedures to clinical response to treatment. Flow cytometry analyses of 726 procedures in 15 patients over at least 6 months were used. Standard ECP procedure was used, with patients undergoing two procedures twice monthly until partial response, followed by monthly procedures until complete response, with response assessed monthly throughout. Analysis showed that CD3^+^ numbers from apheresis in ECP during the early stages of treatment were correlative to subsequent clinical response. This prediction of response may identify patients early on in treatment who are responding to ECP and exclude those who are unlikely to achieve clinical response. Such lymphopenia is indicative of patients with more severe GVHD (126) The corollary of this would be that patients who are responsive to ECP have higher levels of circulating T-cells indicative of less severe GVHD. The distinction between genuine ECP responses from milder forms of GVHD that may resolve spontaneously will need randomized clinical trials. On the other hand, these data may indicate that a minimum dose of, and hematopoietic capability, to supply ECP-treated T-cells is needed to exert therapeutic effect or that the infusion includes circulating allo-reactive T-cell clones (52, 113) More recently, Iniesta et al. (58) reported in 2018 a prospective analysis of 32 GvHD patients undergoing 552 ECP treatments for both, investigating correlation between response to ECP and CD56^bright^ natural killer (NK) cell population. 11 aGvHD and 21 cGvHD patients underwent ECP treatment during a minimum 3-month period, using a standard ECP protocol, with 1–2 procedures every week for 6 weeks, followed by one procedure every 2 weeks for 6 weeks, then one procedure every month until greatest response was seen. Flow cytometry was used to analyze lymphocyte populations from peripheral blood taken before, and at regular intervals throughout ECP treatment. Complete clinical response to ECP, defined as complete resolution of clinical signs and symptoms, was shown to correlate to increased percentages of CD56^bright^ NK cells, or an increased CD56^bright/dim^ ratio. This study demonstrated the change in immune populations to be indicative of better response to ECP, particularly in the first 3 months of treatment and irrespective of GvHD type.

**Table 3:** Summary of biomarkers for response of GvHD to ECP with rationale for candidate biomarker.

**Biomarker**	**Study**	**Pathology**	**Cohort**	**Findings**	**Rationale for candidate biomarker**
Clonal T cells, TCRγ	French et al. (52)	cGvHD	27 HLA-matched allo-BMT patients, 10 without cGVHD and 17 with extensive cGVHD,	Increased circulating clonal T cells showed greater chance of response to ECP	In CTCL, clinical responsiveness to photopheresis has been shown to be dependent on the presence of detectable circulating clonal T cells in the peripheral blood (53)
Immature CD19^+^CD21^−^ B lymphocytes	Kuzmina et al. (54)	cGvHD	49 with moderate (*n* = 25) or severe (*n* = 24) cGVHD	Patients who showed no response to ECP after 6 months had significantly higher proportions of immature CD19^+^CD21^−^ cells prior to ECP	B lymphocytes have been shown to have a role in autoimmune alloimmune diseases such as SLE, and a role in the pathogenesis of cGVHD (54)
mDC, pDC, CD4+, CD8+	Akhtari et al. (55)	cGvHD	25 patients with cGVHD. Data were collected with emphasis on blood cellular markers, clinical response to ECP, and overall survival.	Patients who responded to ECP had higher baseline circulating DCs and T cells	The main objective of the investigation was to elucidate the *in vivo* effect of ECP on numbers of circulating DCs and T cells in patients with cGVHD, which is not well defined (55)
BAFF (B-cell activating factor)	Whittle and Taylor (56)	cGvHD	46 cGVHD patients receiving ECP before and during treatment course	Lower BAFF levels after 1 month of ECP predicted better response at 3 and 6 months	BAFF has roles in immature B-cell survival and promotes production of autoantibodies. Excess BAFF may contribute to cGVHD by protecting alloreactive/ autoreactive clones from apoptosis. Elevated BAFF levels reportedly correlate with cGVHD activity (56)
CD3+	Bertani et al. (57)	cGvHD	Retrospectively assessed 15 cGvHD patients treated for at least 6 months with ECP	CD3+ numbers in early stages of ECP were correlative to subsequent clinical response	The study hypothesized the amount of lymphocytes collected and reinfused during ECP treatment might be associated with response to treatment (57)
CD56^bright^, CD56^bright/dim^ ratio	Iniesta et al. (58)	cGvHD and aGvHD	32 patients with GVHD who underwent 552 ECP procedures	clinical response to ECP correlated to increased percentages of CD56^bright^ NK cells, or increased CD56^bright/dim^ ratio, irrespective of GvHD type	Reduction in the CD56^bright^ NK cell population is associated with cGVHD, could increase in those individuals responding to ECP, and that their longitudinal dynamics may correlate with the grade of response (58)

## Conclusion

**Figure 2:**
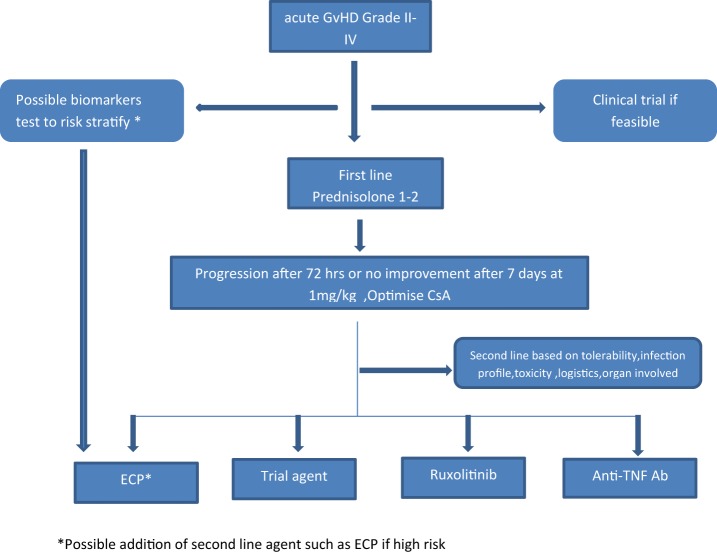
Proposed algorithm for incorporating ECP in the management of aGvHD. *Possible addition of second line agent such as ECP if high risk.

There has been a great increase in recent years in our understanding of the mechanisms underpinning the development of GvHD, its diagnosis and treatment, including mechanisms of ECP. As progress is made from the bench to bedside we can now consider harnessing immunological hallmarks of the condition to develop tests for better and more rapid diagnosis, monitoring and treatment in order to optimize management. In relation to ECP, understanding the immunological basis for the mechanism of action will enable development of robust biomarkers informed algorithms (Figure 2) which will be highly useful in informing patient selection, intensity and duration of the ECP schedule, monitoring of response and decisions regarding combinations with other GvHD therapies. Further research is warranted to establish how GvHD biomarkers are best incorporated in ECP treatment pathways with the goal of tailoring ECP to meet the needs of individual patients and maximizing benefit.

## Author Contributions

All authors contributed equally to the writing of this article, contributed to the drafting, writing, analysis of data, and review prior to submission.

### Conflict of Interest

NM has received grant funding and attended advisory board for Mallinckrodt. JS declares speaker fees from Jazz, Mallinckrodt, Janssen, and Gilead and IDMC membership for a clinical trial funded by Kiadis Pharma. AA has received speaker fees and grant funding from Mallinckrodt. The remaining author declares that the research was conducted in the absence of any commercial or financial relationships that could be construed as a potential conflict of interest.
